# History matching of a complex epidemiological model of human immunodeficiency virus transmission by using variance emulation

**DOI:** 10.1111/rssc.12198

**Published:** 2016-11-24

**Authors:** I. Andrianakis, I. Vernon, N. McCreesh, T. J. McKinley, J. E. Oakley, R. N. Nsubuga, M. Goldstein, R. G. White

**Affiliations:** ^1^ London School of Hygiene and Tropical Medicine UK; ^2^ Durham University UK; ^3^ University of Exeter UK; ^4^ University of Sheffield UK; ^5^ Medical Research Council Uganda Kampala Uganda

**Keywords:** Calibration, Gaussian processes, Individual‐based models, Inverse problems, Stochastic simulators

## Abstract

Complex stochastic models are commonplace in epidemiology, but their utility depends on their calibration to empirical data. History matching is a (pre)calibration method that has been applied successfully to complex deterministic models. In this work, we adapt history matching to stochastic models, by emulating the variance in the model outputs, and therefore accounting for its dependence on the model's input values. The method proposed is applied to a real complex epidemiological model of human immunodeficiency virus in Uganda with 22 inputs and 18 outputs, and is found to increase the efficiency of history matching, requiring 70% of the time and 43% fewer simulator evaluations compared with a previous variant of the method. The insight gained into the structure of the human immunodeficiency virus model, and the constraints placed on it, are then discussed.

## 1 Introduction

Mathematical modelling has played a large role in informing our understanding of infectious disease transmission and epidemiology. In the field of human immunodeficiency virus (HIV), it has been used to investigate the role of partnership concurrency (overlapping sexual partnerships) on HIV transmission (McCreesh *et al*., 2012), to estimate the contribution of acute, early stage infection to overall transmission (Powers *et al*., 2013), and to estimate the proportion of transmission that occurs outside cohabiting partnerships (Bellan *et al*., 2013). Modelling can also be used to inform policy, by allowing the effects of different control interventions to be estimated and compared, without the need for expensive and time‐consuming randomized control trials. For instance, modelling has been used to predict the effects of making antiretroviral therapy universally available to people living with HIV, regardless of how far their disease has progressed (Granich *et al*., 2009), and estimating the effects of expanding access to antiretroviral therapy and/or pre‐exposure prophylaxis in men who have sex with men in the UK (Punyacharoensin *et al*., 2016).

In this study, we analyse a mathematical model of HIV transmission and partnership concurrency, called Mukwano, developed at the London School of Hygiene and Tropical Medicine. It is an individual‐based model with 22 inputs and 18 outputs, and is also stochastic, meaning that repeated evaluations for the same input parameters do not return the same output, but rather samples from a distribution with unknown characteristics. The usefulness of this and other models depends on our ability to calibrate them to measured empirical data (Grimm *et al*., 2006; May, 2004). Calibration is a type of inverse problem that attempts to estimate the input parameters of a system, such that its outputs are consistent with the available empirical data. Poor calibration results in a mathematical model that does not accurately reflect what we know about the current situation, greatly reducing our ability to make projections into the future. Poor calibration can also result in the amount of uncertainty in future projections being underestimated, leading to overconfident predictions being made, and potentially harmful policy decisions.

Calibration approaches range from simple least squares estimation techniques to advanced probabilistic methodologies. Markov chain Monte Carlo based techniques (Gibson and Renshaw, 1998; O'Neill and Roberts, 1999) are popular calibration methodologies. However, they tend to require the calculation of the likelihood function, which in the case of Mukwano is not available, whereas a data augmentation approach would require a numerical integration over a very large hidden state space. In smaller‐scale models, simulation‐based techniques, based on repeated evaluations of the simulator (Toni *et al*., 2009; McKinley *et al*., 2009; Andrieu *et al*., 2010) have been applied with some success. The simulator that we are analysing in this work has a large number of inputs and outputs, and would require a large number of evaluations because of

- the high dimensionality of the input space and
- the part of that space that matches the empirical data can be very small, because of the multiple constraints that are imposed by the large number of outputs.

Furthermore, Mukwano is a stochastic simulator, which requires multiple evaluations for each set of inputs to extract statistics about its output values, such as means and variances. Finally, the above methods attempt to make inferences over the entire input space by using all available outputs at once. This can be an unnecessarily complicated task, as it requires simultaneously capturing the behaviour of all outputs in parts of the input space that are very far from the ‘region of interest’.

History matching (Craig *et al*., 1997) is a form of calibration methodology, which is sometimes referred to as a precalibration step, that is designed to address the above problems. It is based on the use of an ‘emulator’, which is a statistical model of the simulator that is fast to evaluate (Sacks *et al*., 1989), and is therefore less disadvantaged by long simulator running times. It works by rejecting the input space where the simulator does not match the data, rather than the other way round. As a result, the entire set of outputs does not have to be taken into account at once, thus reducing the burden of analysing a large number of possibly complex outputs simultaneously, as is required by more traditional approaches. Finally, it focuses in on the region of interest in a series of iterations (waves), bypassing the need to model all of the simulator's outputs over all of its input space, and benefitting from the fact that Mukwano is expected to be ‘well behaved’ in smaller input space regions. These characteristics make history matching particularly suitable as a precalibration step for simulators with large numbers of inputs and outputs, and long evaluation times which make the direct application of other calibration methodologies nearly impossible. Additionally, it may be viewed as an appropriate analysis methodology for simulators that are not considered to be sufficiently accurate to warrant a full Bayesian analysis, which is much more computationally expensive.

History matching is not only useful for producing a large number of calibrated input samples. A careful study of the patterns that appear in the input and output spaces can be very informative about the way that the simulator models various processes as well as the effect that the constraints imposed by the empirical data have on the structure of the non‐implausible space. These features of history matching illustrate the way that Mukwano handles the HIV transmission process and how the empirical data shape the values that input parameters, such as contact rates and concurrency parameters, are allowed to take.

In previous work (Andrianakis *et al*., 2015), history matching was applied to Mukwano, but a rough approximation was used to account for the stochastic variability in its outputs, which was found to slow the method's convergence unnecessarily. In the present work, we refine the treatment of stochastic outputs by explicitly emulating their variance in addition to their mean and improving the overall efficiency of history matching. We also study correlation patterns between calibrated input and output samples, which provide useful insights into various processes of the simulator.

The structure of this paper is as follows: Section 2 describes the stochastic simulator that is studied, which is a dynamic, individual‐based HIV simulator, calibrated with epidemiological and behavioural data from a cohort in Uganda. Section 3 describes history matching in its standard form and introduces the proposed adaptation that allows it to handle stochastic models. Section 4 presents the results of history matching with the proposed adaptation, and Section 5 concludes the paper.

The data that are analysed in the paper and the programs that were used to analyse them can be obtained from

http://wileyonlinelibrary.com/journal/rss-datasets

## 2 Model description and problem set‐up

The simulator that we analyse in this work, which is known as Mukwano, is a dynamic, stochastic, individual‐based computer model that simulates heterosexual sexual partnerships and HIV transmission (McCreesh *et al*., 2012). Each individual is represented by a number of characteristics, of which some remain constant during the simulated life (e.g. gender and date of birth), whereas others can change (e.g. HIV status). Changes in personal characteristics result from events such as the start and the end of sexual relationships. These events are stochastic: if and when an event occurs is determined by sampling from appropriate probability distributions. To generate model outcomes for a simulated population, the characteristics of the simulated individuals are aggregated.

Births, deaths, partnership formation and dissolution, and HIV transmission are modelled by using time‐dependent rates. At birth, simulated individuals are assigned to one of two sexual activity groups (‘high activity’ and ‘low activity’), and to one of two partnership concurrency groups (‘high concurrency’ and ‘low concurrency’). Each sexual activity group has associated male and female sexual contact rates, which determine the rate at which individuals form new partnerships. The duration of each new partnership is determined by the activity group of one of the partners, chosen at random. If their activity group is high, the partnership will have a short duration. If it is low, the partnership will have a long duration.

**Figure 1:**
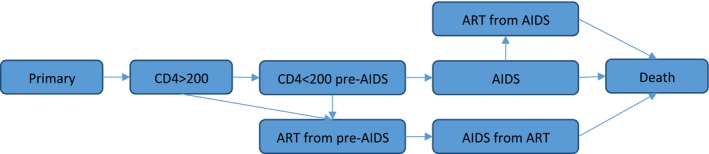
Schematic diagram of simulated HIV natural history and antiretroviral (‘ART’) treatment

Seven different HIV stages are simulated, as shown in Fig. 1. The natural history of HIV before antiretroviral therapy is represented by four stages: primary, CD4 cell count 200 cells *μ*l^−1^ or greater, CD4 cell count less than 200 cells *μ*l^−1^ before acquired immune deficiency syndrome (AIDS) diagnosis and AIDS. Infected people move sequentially through the four stages, and each stage has an associated HIV transmission probability. After 2004, when antiretroviral therapy first became available in the population that we are modelling, simulated people can be in an additional three stages: antiretroviral therapy from pre‐AIDS, antiretroviral therapy from AIDS, and AIDS from antiretroviral therapy. Possible routes of progression through the seven stages are shown in Fig. 1. The probability of moving from a non‐antiretroviral‐therapy stage to an antiretroviral therapy stage increased between 2004 and 2008, representing the increasing availability of antiretroviral therapy in the population over time.

20 behavioural and two epidemiological inputs are varied, including a mixing parameter, which determines the tendency for individuals to form partnerships preferentially with people in their own activity group, and an input which determines the duration of the long and short duration partnerships. Many behavioural inputs are permitted to take different values in each of three calendar time periods. This enables sexual behaviour to vary over time and allows the simulator to be fitted to trends in HIV prevalence in the population. A full list of the 22 simulator inputs and their original plausible ranges is shown in Table 1.

**Table 1:** Simulator input parameter description and ranges†

*Number*	*Input description*	*Abbreviation*	*Minimum*	*Maximum*
1	Proportion of men in the high sexual activity group	mhag	0.01	0.5
2	Proportion of women in the high sexual activity group	whag	0.01	0.5
3	Mixing by activity group (*ε*)	mag	0	1
4	High activity contact rate (risk behaviour 1) (partners per year)‡	hacr1	0	10
5	Low activity contact rate (risk behaviour 1) (partners per year)‡	lacr1	0	2
6	Start year for risk behaviour 2	sy2	1986	1992
7	High activity contact rate (risk behaviour 2) (partners per year)‡	hacr2	0	10
8	Low activity contact rate (risk behaviour 2) (partners per year)‡	lacr2	0	2
9	Start year for risk behaviour 3	sy3	1998	2002
10	High activity contact rate (risk behaviour 3) (partners per year)‡	hacr3	0	10
11	Low activity contact rate (risk behaviour 3) (partners per year)‡	lacr3	0	2
12	Mean HIV transmission probability per sex act during primary stage of infection (mean of male‐to‐female and female‐to‐male transmission probabilities)	atp	0	1
13	Ratio of male‐to‐female/female‐to‐male transmission probabilities	rtp	1	3
14	Proportion of low activity men in high concurrency group	lmhc	0	1
15	Proportion of low activity women in high concurrency group	lwhc	0	1
16	Male concurrency parameter in high concurrency group (risk behaviour 1)	mchc1	0	1
17	Female concurrency parameter in high concurrency group (risk behaviour 1)	fchc1	0	1
18	Male concurrency parameter in high concurrency group (risk behaviour 2)	mchc2	0	1
19	Female concurrency parameter in high concurrency group (risk behaviour 2)	fchc2	0	1
20	Male concurrency parameter in high concurrency group (risk behaviour 3)	mchc3	0	1
21	Female concurrency parameter in high concurrency group (risk behaviour 3)	fchc3	0	1
22	Duration of long duration partnerships (years)	dlp	5	20

†These define the input parameter space over which the history match search is performed. ‡Simulator input parameters that codetermine partnership formation. The actual rate of partnership formation in the simulator will vary from this because of adjustment for concurrency and partnership balancing.

The simulator is calibrated to 18 demographic, behavioural and epidemiological outputs that include male and female population sizes in 2008, and male and female HIV prevalences at three time points. They also include some outputs that ensure that the prevalence and incidence of monogamous and concurrent sexual partnerships in the simulator closely match estimates from the empirical population. The empirical data were collected from a rural general population cohort in south‐west Uganda. The cohort was established in 1989 and currently consists of the residents of 25 villages (Mulder *et al*., 1994a,b; Seeley *et al*., 1991). Every year, demographic information on the cohort is updated, the population is tested for HIV and a behavioural questionnaire is conducted. In 2008, this included questions that allowed the prevalence of monogamous and concurrent short duration and long duration partnerships to be estimated. All 18 simulator outputs and their empirical data are shown in Table 2. The intervals that are given for each of the outputs represent the limits for an acceptable match, and we considered them to be 95% confidence intervals for the purposes of the calibration. Their mean value was therefore used to define the value of the empirical data **z**, and their difference was considered to represent four times the square root of the observation error variance *V*_o_.

**Table 2:** Description of simulator outputs and the limits defined as an acceptable match

*Number*	*Output description*	*Abbreviation*	*Minimum*	*Maximum*
1	Population size in 2008 (male)	psm	2986	3650
2	Population size in 2008 (female)	psf	3374	4124
3	Average male partnership incidence in 2008 (partners per year)	ampi	0.4	0.489
4	HIV prevalence in 1992 (male)	p92m	0.084	0.112
5	HIV prevalence in 1992 (female)	p92f	0.096	0.124
6	HIV prevalence in 2001 (male)	p01m	0.07	0.09
7	HIV prevalence in 2001 (female)	p01f	0.083	0.107
8	HIV prevalence in 2007 (male)	p07m	0.06	0.084
9	HIV prevalence in 2007 (female)	p07f	0.093	0.119
10	Point prevalence of men with 1 long duration partnership in 2008 (%)	m1l	34.62	42.31
11	Point prevalence of men with 1 short duration partnership in 2008 (%)	m1s	10.86	13.27
12	Point prevalence of men with 1 partnership (either type) in 2008 (%)	m1	37.83	46.24
13	Point prevalence of men with 2 or more long duration partnerships in 2008 (%)	m2l	3.38	4.13
14	Point prevalence of men with 2 or more short duration partnerships in 2008 (%)	m2s	1.69	2.07
15	Point prevalence of men with 2 or more partnerships (any combination) in 2008 (%)	m2	8.66	10.59
16	Point prevalence of women with 2 or more long duration partnerships in 2008 (%)	w2l	0.85	1.03
17	Point prevalence of women with 2 or more short duration partnerships in 2008 (%)	w2s	0.42	0.52
18	Point prevalence of women with 2 or more partnerships (any combination) in 2008 (%)	w2	2.17	2.65

As mentioned previously, the simulator is stochastic, and the variance of the outputs changes, sometimes drastically, with changes in the values of the input parameters. In Andrianakis *et al*. (2015), it was observed that not accounting properly for the variance in the outputs, and in particular their dependence on the input values, reduced the efficiency of history matching and limited the insight that was gained into Mukwano's structure and the consequences of the observational constraints. The methodological developments that are proposed in this work address this issue.

## 3 Methods—history matching

### 3.1 Overview

History matching is a method that attempts to identify the part of the simulator's input space that is likely to result in matches between the simulator's outputs and the empirical data (observations). This part of the input space is referred to as *non‐implausible* and has a high probability of containing the vast majority of the input parameters’ posterior mass. This space's complement is known as *implausible*, where matches between the outputs and the observations are highly unlikely to be found. History matching was first developed in the field of oil reservoir simulations (Craig *et al*., 1997) but has since been applied to the calibration of computer models in fields ranging from galaxy formation, oceanography, systems biology and epidemiology (Vernon *et al*., 2010a, 2014; Vernon and Goldstein, 2010; Goldstein *et al*., 2013; Williamson *et al*., 2013; Andrianakis *et al*., 2015).

History matching works in iterations, known as *waves*, where the implausible space is first identified and then discarded. Each wave focuses the search for implausible space in the space that was characterized as non‐implausible in all previous waves; thus, the non‐implausible space shrinks with each iteration. The implausibility of the input space is determined with the *implausibility measure*, which is a measure of the distance between the observations and the simulator's output when evaluated at input **x**.

Even though the implausibility measure could be calculated by using the simulator directly, this turns out to be impractical even for simulators of moderate complexity. The reason is that the input space is high dimensional, and an exhaustive search would require a prohibitively large number of simulator evaluations. For this reason, fast surrogates of the simulator are used, which are known as *emulators*. An emulator is essentially a regression model, that predicts the simulator's output for a particular input **x** and is also capable of quantifying the uncertainty of these predictions. A key feature of an emulator is its almost negligible evaluation time. Gaussian processes (GPs) are used to build the emulators in this work; however, Bayes linear models (Vernon and Goldstein, 2010) or simpler substitutes such as linear regression models could also be employed.

### 3.2 History matching of stochastic simulators (fixed variance)

In this section, we present a summary of history matching of stochastic simulators, as presented in Andrianakis *et al*. (2015). The next section introduces the extensions to history matching that improve its efficiency on the calibration of Mukwano and stochastic simulators in general.

We suppose that the simulator has *P* inputs denoted as **x**=(*x*_1_,*x*_2_,…,*x*_*P*_)^T^, which are continuous and lie in a bounded subset $X\subset R^{P}$. The simulator also has *R* outputs *f*(**x**), the *r*th of which is denoted by *f*_*r*_(**x**). Suppose also the existence of **z**=(*z*_1_,*z*_2_,…,*z*_*R*_)^T^ observations, one for each simulator output, which typically come with their own uncertainty bounds (e.g. 95% confidence intervals).

Unlike deterministic simulators, which return the same value each time that they are evaluated at the same input **x**, stochastic simulators typically return draws from a distribution, which has a mean and a variance that we respectively denote by *g*(**x**) and *s*(**x**). We write the *r*th output of the *k*th evaluation of a stochastic simulator at input **x** as

(1) $$f_{r,\;k}\left(x\right)=g_{r}\left(x\right)+\epsilon_{r,\;k}\left(x\right),$$

where *g*_*r*_(**x**) is the mean and *ε*_*r*,*k*_(**x**) is a zero‐mean noise term with variance *s*_*r*_(**x**).

At each wave, the simulator is evaluated *K* times at each of the *N* design points; an estimate of the mean simulator's response is

(2) $${\hat{g}}_{r}\left(x_{n}\right)=\frac{1}{K}\sum_{k=1}^{K}f_{r,\;k}\left(x_{n}\right).$$

An emulator of the simulator's mean output is then built by using the training points $D=\{x_{n},{\hat{g}}_{r}\left(x_{n}\right)\}$, for all the outputs that this is possible, which at wave *η* are denoted as $r\in R_{\eta }$. We write the emulator's prediction for the mean output as *E* ^*^[*g*_*r*_(**x**)] and the uncertainty of the prediction (variance) as *V*_*c*,*r*_(**x**).

An estimate of the simulator's variance at each of these points is

(3) $${\hat{s}}_{r}\left(x_{n}\right)=\frac{1}{K-1}\sum_{k=1}^{K}{\left\{f_{r,\;k}\left(x_{n}\right)-{\hat{g}}_{r}\left(x_{n}\right)\right\}}^{2}.$$

Andrianakis *et al*. (2015) used the 90th percentile of the variances ${\hat{s}}_{r}\left(x_{n}\right)$, *n*=1,…,*N*, which we denote by *V*_*s*,90_, in the calculation of the implausibility measure.

The implausibility measure for the *r*th output is formulated as the distance between *z*_*r*_ and *E* ^*^[*g*_*r*_(**x**)], weighted with the uncertainty that is introduced by the error terms that link the two quantities.

We assume that *z*_*r*_ is a noisy measurement from an underlying, unobservable physical process *y*_*r*_, with their relationship described by

(4) $$z_{r}=y_{r}+\phi_{r},$$

where *ϕ*_*r*_ is a random variable that follows a unimodal distribution with zero mean and variance *V*_o_,_*r*_. Its variance can be derived from considerations of the measurement process and, as it links the (unobserved) physical process *y*_*r*_ and the measurements *z*_*r*_, has no dependence on the simulator's inputs **x**.

The physical process *y*_*r*_ is linked to a single realization of the simulator *f*_*r*,*k*_(**x**) via the model discrepancy *δ*_*r*_, using

(5) $$y_{r}=f_{k,\;r}\left(x^{*}\right)+\delta_{r},$$

where **x** ^*^ is known as the ‘best input’. This discrepancy arises because simulators are virtually always simplifications of the physical process *y*_*r*_ (reality), either because we do not fully understand *y*_*r*_, and therefore cannot model it exactly, or because some parts have been deliberately left out of the modelling process. Accounting for model discrepancy can protect against overfitting the (potentially) wrong values of **x** and makes the simulator's predictions more robust. For more on this point the reader can consult Kennedy and O'Hagan (2001), Goldstein and Rougier (2009), Vernon *et al*. (2010a) and Brynjarsdottir and O'Hagan (2014). The *δ*_*r*_‐term represents the model expert's beliefs about the simulator's deficiencies and as such is subjective and should be treated with caution. Methods for a structured elicitation of model discrepancy were discussed in Goldstein and Rougier (2009) and Goldstein *et al*. (2013). In Andrianakis *et al*. (2015), as well as in the present work, *δ*_*r*_ is assumed to follow a unimodal distribution with zero mean and variance *V*_*m*,*r*_; as *δ*_*r*_ is defined as the difference between *y*_*r*_ and the simulator evaluated at its ‘best value’ **x** ^*^, it is also **x** invariant.

Finally, because we are using an emulator in place of the actual simulator, we need to take into account the error between the emulator's prediction *E* ^*^[*g*_*r*_(**x**)] and the simulator's mean output *g*_*r*_(**x**), which we denote by *ζ*_*r*_(**x**)=*g*_*r*_(**x**)−*E* ^*^[*g*_*r*_(**x**)]. *ζ*_*r*_(**x**) is also assumed to be unimodal with zero mean and variance *V*_*c*,*r*_(**x**).

Combining the above with equations (1), (4) and (5) the link between the observed data *z*_*r*_ and the emulator's prediction *E* ^*^[*g*_*r*_(**x**)] is

$$z_{r}=E^{*}\left[g_{r}\left(x^{*}\right)\right]+\phi_{r}+\delta_{r}+\zeta_{r}\left(x^{*}\right)+\epsilon_{k,\;r}\left(x^{*}\right).$$

On the basis of the above analysis, the implausibility measure for a single output *r* at a given value of **x** is given by

(6) $$I_{r}\left(x\right)=\frac{|z_{r}-E^{*}\left[g_{r}\left(x\right)\right]|}{{\left\{V_{o,\;r}+V_{m,\;r}+V_{c,\;r}\left(x\right)+s_{r}\left(x\right)\right\}}^{1/2}}.$$

Equation (6) is a measure of the distance between the observation *z*_*r*_ and the emulator's posterior mean *E* ^*^[*g*_*r*_(**x**)], weighted by the square root of the variances of all the uncertainties that we have considered so far.

In general, *s*_*r*_(**x**) is unknown unless the simulator is evaluated at **x**. In Andrianakis *et al*. (2015) this was approximated by *V*_*s*,90_, the 90th percentile of the observed variances, which could be seen as a conservative estimate in the absence of more detailed information. The approximation of *s*_*r*_(**x**) with *V*_*s*,90_ is clearly rough, because it essentially assumes that the variance of the simulator's output is constant with respect to the input **x** (fixed variance), which is not necessarily true. As a result, the rejection of input space is not as efficient as it would have been if more accurate estimates of the variance were available. In the present work, we aim to refine this approximation via the use of variance emulators. This will be described in Section 3.3.

The relaxed distributional assumptions that we made for the uncertainty terms *ϕ*,* δ* and *ζ* (i.e. simply that they have zero mean and are unimodal) allow the use of Pukelsheim's 3*σ*‐rule (Pukelsheim, 1994) to derive cut‐off limits for the above implausibility measure, such that, when the value of *I*_*r*_(**x**) is larger than a cut‐off *I*_*c*_, then the input **x** can be considered implausible. Pukelsheim's 3*σ*‐rule is a powerful (and underused) result which states that any continuous unimodal distribution has at least 95% of its probability mass within 3 standard deviations, regardless of any asymmetry in the distribution, i.e. if **x** were indeed the best input **x** ^*^, then *I*(**x**) should be 3 or less with probability 95% for even the most asymmetric (but still unimodal) distributions that contribute to *I*(**x**), and with a probability that is much higher than 95% for more symmetric, less unusual cases. Therefore, if *I*_*r*_(**x**)>3 it suggests that we would be unlikely to obtain an acceptable match between outputs and observed data, if we were to run the simulator at **x** (see Vernon *et al*. (2010a) for details). We note here that the parts of the input space for which *I*_*r*_(**x**)<3 do not necessarily lead to matches between *z*_*r*_ and *g*_*r*_(**x**), and hence do not necessarily represent a ‘good’ input **x**: the implausibility can be small either because *z*_*r*_ and *E* ^*^[*g*_*r*_(**x**)] are close, or because there is still a large amount of uncertainty regarding the simulator's behaviour at **x**. In other words, the denominator of *I*_*r*_(**x**) is still large.

The above single‐output implausibility measure has natural extensions to several outputs. One such extension is the maximum implausibility defined as

(7) $$I_{M}\left(x\right)=\max_{r\in R_{\eta }}\left\{I_{r}\left(x\right)\right\},$$

where *R*_*η*_ is the set of outputs that we wish to consider in wave *η*. Further extensions and analysis on implausibility measures can be found in Vernon *et al*. (2010a). Note that this definition involves only a subset of the outputs as represented by the set *R*_*η*_. Often, at early waves of the history match we would only emulate and construct implausibility measures for a small subset of the outputs, as some outputs may be very badly behaved over the whole input space. This subset *R*_*η*_ would usually increase in size in later waves as we narrow the search to a smaller region of input space. This should be compared with a standard fully Bayesian or likelihood‐based analysis, where one has the difficult task of modelling all outputs simultaneously from the outset. This is a major strength of history matching.

### 3.3 History matching with variance emulation

In the previous section, we claimed that using a fixed estimate for the simulator's variance reduces the efficiency of history matching, because it assumes that *s*_*r*_(**x**) is constant with respect to **x**, which is not true in general. In this section, we propose a method that mitigates this problem by providing better estimates of the variance via emulation. The method is based on an independent emulation of the mean *g*(**x**) and variance *s*(**x**). At each wave, the simulator is evaluated at *N* points and the training data $\left\{x_{n},\hat{g}\left(x_{n}\right)\right\}$ and $\left\{x_{n},\hat{s}\left(x_{n}\right)\right\}$ are calculated by using equations (2) and (3). We start with a description of the emulator of the mean.

A GP emulator is built by considering a GP as a prior for the simulator's *r*th output:

(8) $$g_{r}\left(x\right)∼N\left\{h\left(x\right)\beta ,\sigma^{2}c\left(x,x^{\prime }\right)\right\}.$$

The GP has a mean function *h*(**x**)***β***, with *h*(**x**) being a vector of deterministic functions of **x** (regressors), ***β*** a vector of regression coefficients and *σ* ^2^ the variance of the process. The correlation function *c*(**x**,**x′**) can be a kernel, such as a Gaussian or a Matérn kernel, that determines the correlation between *g*_*r*_(**x**) and *g*_*r*_(**x**&#*x*2019;).

A key point here is that the training data $D_{m}\equiv \left\{x_{n},\hat{g}\left(x_{n}\right)\right\}$ are not the actual mean outputs of the simulator, but estimates, the accuracy of which depends on the variance *s*(**x**_*n*_) and the number of repetitions *K*. For this reason, we use a heteroscedastic noise component in the emulators of the mean, i.e. a noise term that will be different for each training point. The GP model of the training data is

$$g_{r}\left(D_{m}\right)∼N\left[H\beta ,\sigma^{2}A+\text{diag}\left\{{\left(\nu_{1},\nu_{2},\ldots ,\nu_{n}\right)}^{T}\right\}\right].$$

In this equation, *H* is an *n*×*q* matrix, whose *n*th row is the polynomial *h*(**x**_*n*_) from equation (8). *A* is a symmetric correlation matrix with entries *A*_*i*,*j*_=*c*(**x**_*i*_,**x**_*j*_). The noise components *ν* are calculated as $\nu_{n}={\hat{s}}_{n}\left(x_{n}\right)/K$ and the operator diag(·) transforms the column vector into a diagonal matrix.

The hyperparameters in the above expression are estimated by using maximum likelihood (for example see Andrianakis *et al*. (2015) and Rasmussen and Williams (2006)) and the above emulator provides an estimate of the simulator's mean value at an untested point **x** and an associated variance of the estimate. We denote these two quantities as *E* ^*^[*g*_*r*_(**x**)] and *V*_*c*,*r*_(**x**). All emulators are validated by using a separate validation set of simulator runs, following the methods that were described in Bastos and O'Hagan (2009).

A similar procedure is followed for emulating the variances. First, we log‐transform the variance data defining $\hat{\xi }\left(x\right)\equiv \text{ln}\left\{\hat{s}\left(x\right)\right\}$, resulting in the training data set $D_{v}=\left\{x_{n},\hat{\xi }\left(x_{n}\right)\right\}$. This transformation is helpful, because $\hat{\xi }\left(x\right)$ is closer to a Gaussian distribution than $\hat{s}\left(x\right)$ is, and therefore easier to model by using a GP. The prior for the training data is

$${\hat{\xi }}_{r}\left(D_{v}\right)∼N\left(H\beta ,\sigma^{2}A+I\nu \right).$$

Since the data *D*_v_ are also estimates we could have used the same heteroscedastic model as we used for the emulators of the mean. However, this would require estimating the *ν*s by using a fourth‐order statistic of the simulator runs (variance of the variance), which could be unstable unless we had a very large number of repetitions per design point. For this reason, we take a simpler approach and assume that the noise level in the variance data is constant and equal to *ν*, which is a hyperparameter that is estimated along with the other hyperparameters of the GP, using maximum likelihood.

The variance emulators provide an estimate of the log‐variance for any untried input **x** in the current non‐implausible space, which we denote by *E* ^*^[*ξ*_*r*_(**x**)] and will be used in the implausibility that is defined in Section 3.3.1. Variance emulation in a regression setting has also been discussed in Henderson *et al*. (2009), Vernon and Goldstein (2010), Ankenman *et al*. (2010) and Boukouvalas *et al*. (2014), but its integration within the history matching framework is studied here for the first time.

#### 3.3.1 The implausibility measure (emulated variance)

The implausibility measure for one output is again given by equation (6). In this case, the variance emulators provide an improved approximation to *s*_*r*_(**x**), which is *s*_*r*_(**x**)≈ exp {*E* ^*^[*ξ*(**x**)]}. This term is a function of **x** and should therefore be more accurate than the fixed *V*_*s*,90_ as used previously. Furthermore, in most cases it should hold that  exp {*E* ^*^[*ξ*(**x**)]}<*V*_*s*,90_, which results in larger implausibility values for a given **x** and, therefore, a more efficient space rejection. The proposed implausibility measure for one output takes the form

(9) $$I_{r}\left(x\right)=\frac{|z_{r}-E^{*}\left[g_{r}\left(x\right)\right]|}{{\left[V_{o,\;r}+V_{m,\;r}+V_{c,\;r}\left(x\right)+\text{exp}\left\{E^{*}\left[\xi_{r}\left(x\right)\right]\right\}\right]}^{1/2}}.$$

**Figure 2:**
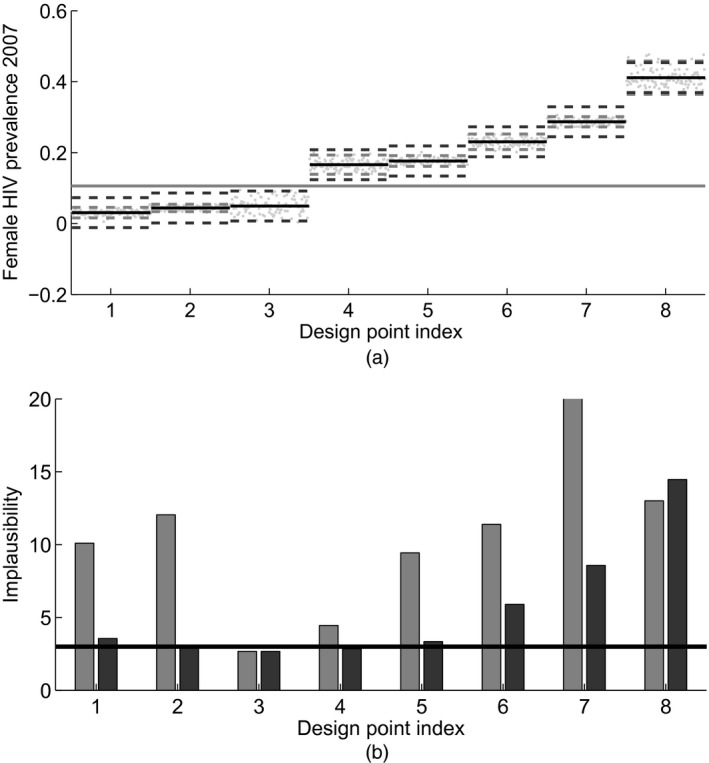
Improvements due to the emulated variance method: (a) 100 simulator evaluations in eight different design points (), their mean (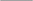) and ±2 standard deviations calculated with *V*_*s*,90_ (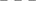) or with the actual variance calculated by the 100 repetitions at each design point (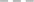) (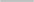, mean of the empirical data); (b) simplified form of the implausibility calculated with *V*_*s*,90_ (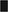) or with the actual variance (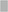) (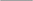, implausibility cut‐off)

This argument is illustrated in Fig. 2. Fig. 2(a) shows 100 simulator evaluations at each of eight design points (grey dots). The output that is studied is the 2007 female HIV prevalence. The horizontal black lines show the estimated mean of each design point, and the black broken lines represent ±2 standard deviations calculated with the second largest variance of the design points shown (similar to *V*_*s*,90_). The broken grey lines show ±2 standard deviations, calculated with the variance estimated from the actual 100 repetitions at each design point. The observations are shown with the horizontal grey line. The bars in Fig. 2(b) show a simplified form of the implausibility $I=|z-\hat{g}\left(x\right)|/\hat{s}{\left(x\right)}^{1/2}$, calculated with the respective variances of Fig. 2(a). The horizontal black line is the cut‐off implausibility value, which is set at 3. Fig. 2 shows that the overestimation of the variance for points 1, 2, 4 and 5 by the use of *V*_*s*,90_ reduces their implausibility, such that they are either accepted or rejected marginally (*I*(**x**)≈3). Improving the estimate of *s*(**x**) increases the implausibility and allows rejecting those points with greater confidence. This toy example conveys the essence of the method that we are proposing in this work.

#### 3.3.2 Procedure

The procedure of history matching by using the emulated variance is presented below.

- Define the initial *P*‐dimensional non‐implausible space $X_{\eta =0}$.
- Select *N* training and *N*′ validation points from the current non‐implausible space $X_{\eta }$, using a space filling design.
- Evaluate the simulator *K* times at each of the training and validation points; calculate the training data *D*_m_ for the mean and *D*_v_ for the variance.
- Build and validate an emulator for as many of the *g*_*r*_(**x**) as possible.
- Build and validate an emulator for as many of the *ξ*_*r*_(**x**) as possible.
- Evaluate the implausibility measure *I*_M_(**x**) for a large number of $x\in X_{\eta }$ such that the complexity of $X_{\eta }$ is represented with sufficient accuracy. Use the single‐output implausibility from equation (9). $X_{\eta +1}$ is the set of $x\in X_{\eta }$ for which *I*_M_(**x**) is less than the chosen threshold.
- Increase wave counter *η* by 1 and repeat steps (b)–(f), until
  - the emulator uncertainty *V*_*c*_ is smaller than the other uncertainties (e.g. *V*_o_ or *V*_m_), so more waves would not reduce $X_{\eta }$ further, or
  - a large number of simulator runs from the final wave's non‐implausible space are sufficiently close to the observations for the needs of the application or
  - all $X_{\eta }$ have been characterized as implausible.

In the above sequence of non‐implausible spaces, it holds that $X_{\eta }\subset X_{\eta -1}\subset \;\;\ldots \subset X_{0}$. During this process of space reduction, $X_{\eta }$ might lose properties such as convexity or connectivity. In general, history matching can handle non‐convex spaces, as it can identify, for example, disconnected regions. Ideally, if disconnected regions were to be found in $X_{\eta }$, they could be emulated separately. In most cases, however, identifying such regions in high dimensional spaces is far from trivial. As for the GP emulators, these can be thought of as defined over the wider (convex) region, but we choose only to evaluate them, for history matching, within the non‐implausible space. For more on this point the reader can consult Vernon *et al*. (2010b).

History matching is also very efficient in dealing with models that are unidentifiable. Correlation ridges and multiple modes in the posterior, typical manifestations of identifiability issues, pose no problem to history matching, whereas they can plague other methods, including methods based on Markov chain Monte Carlo sampling. Finally, if the simulator is incapable of matching the observations, history matching will reject all the input space as implausible, flagging this condition, whereas other likelihood‐ and simulation‐based methods will always attempt to return a posterior distribution, regardless of how poorly the simulator fits the data.

#### 3.3.3 Further points—extensions

The value of *N* can be determined by the available computational resources, but a very rough rule of thumb suggests setting *N*=10*P*, where *P* is the number of inputs (Loeppky *et al*., 2009). The number of validation runs can be chosen as *N* ^′^≈*N*/10. The training and validation data are best selected by using some space filling method, e.g. by maximizing the minimum distance between points, such that they fill the entire input space. This type of design generally leads to emulators that can more accurately describe the simulators over most parts of the non‐implausible space. Furthermore, simulator runs from previous waves can be used as training points for the present wave if they fall within or close to the current non‐implausible region.

The number of repeated evaluations *K* of the simulator at each design point is considered fixed throughout this work. The value of *K* can be chosen such that the variance of the estimator $\hat{g}\left(x\right)$, which is given by $\hat{s}\left(x\right)/K$, is smaller than the observation error, and also by considering the computational budget that is available for running the simulator. Another approach would be to use a variable number of repetitions *K*(**x**), such that the simulator is evaluated more times at the **x**s where *s*(**x**) is expected to be large, and vice versa. The emulators of the variance can provide some guidance in this direction, as they can predict *s*(**x**) at a new location **x**. This possibility has been explored in a kriging setting in Fedorov and Hackl (1997), Ankenman *et al*. (2010) and in an optimization setting in Picheny *et al*. (2013). Although using a fixed number of repetitions is a simple and robust approach to the problem, a *K* that varies with the input **x** could further increase the efficiency of history matching by reducing the total number of simulator evaluations.

The simulator's 18 outputs were modelled with independent univariate emulators. Note that it is the ‘residual processes’ *g*(**x**)−*h*(**x**)***β*** that are assumed to be independent between the different outputs. The independent emulators can therefore still capture strong correlations between outputs via the trend term *h*(**x**)***β***, which often justifies putting more detail into it, as is discussed in Vernon *et al*. (2010a, 2014). Nevertheless, it is possible that the efficiency of history matching could be improved by using multivariate emulators if there are correlations between these residual processes. Emulators with separable covariance functions such as those in Rougier (2008) or Conti and O'Hagan (2010) could be used, although these require the same set of correlation length parameters to be used for each output. Multivariate emulators with non‐separable covariance functions were discussed in Fricker *et al*. (2013), but these are more computationally demanding to fit.

## 4 History matching the Mukwano simulator

### 4.1 Comparison with the fixed variance approach

To evaluate the benefits of including the variance emulators in history matching, we compare it with the history match that was shown in Andrianakis *et al*. (2015). In that work, the *fixed variance* approach was used, i.e. the variance of the simulator's output *s*(**x**) was not emulated, but was rather fixed to the 90th quantile of the estimated variances $\left\{\hat{s}\left(x_{n}\right):n=1,\ldots ,N\right\}$. In the *emulated variance* methodology proposed, the variance in the output of the stochastic model is emulated and is therefore a function of the input **x**.

To facilitate the comparison, both history matches were designed to be the same in terms of the number of simulator runs per wave, the number of simulator inputs and outputs, and the empirical data. The observation variance terms *V*_o_ were also identical, and the model discrepancy was set in both cases equal to 10% of the variance of $\left\{\hat{g}\left(x_{n}\right)\right\}$, *n*=1,…,*N*. Since the emulated variance approach essentially estimates *s*_*r*_(**x**) as a function of **x**, instead of using a fixed and relatively large value, we expect that the efficiency of history matching will increase. In what follows, we show that this is indeed so, and we demonstrate that the benefits that are gained by the inclusion of the variance emulators outweigh the extra computational effort of building them. A comparison of the costs in terms of central processing unit and user time is given in Section 4.3.

To quantify the closeness of an actual simulator run to the empirical data **z**, we define the *simulator run implausibility* for a single output as

(10) $$I_{R,\;r}\left(x\right)=\frac{|z_{r}-{\hat{g}}_{r}\left(x\right)|}{{\left\{V_{o,\;r}+V_{m,\;r}+{\hat{s}}_{r}\left(x\right)\right\}}^{1/2}}.$$

Note that no emulators are involved in this metric, and it is not part of the history matching algorithm; it is only a metric that quantifies how close the simulator's output is to the empirical data, when evaluated at input **x**. The *overall* simulator run implausibility is defined as the maximum of $I_{R,\;r}\left(x\right)$ across all outputs, i.e. $I_{R}\left(x\right)=\;\max_{r}\left\{I_{R,\;r}\left(x\right)\right\}$.

**Figure 3:**
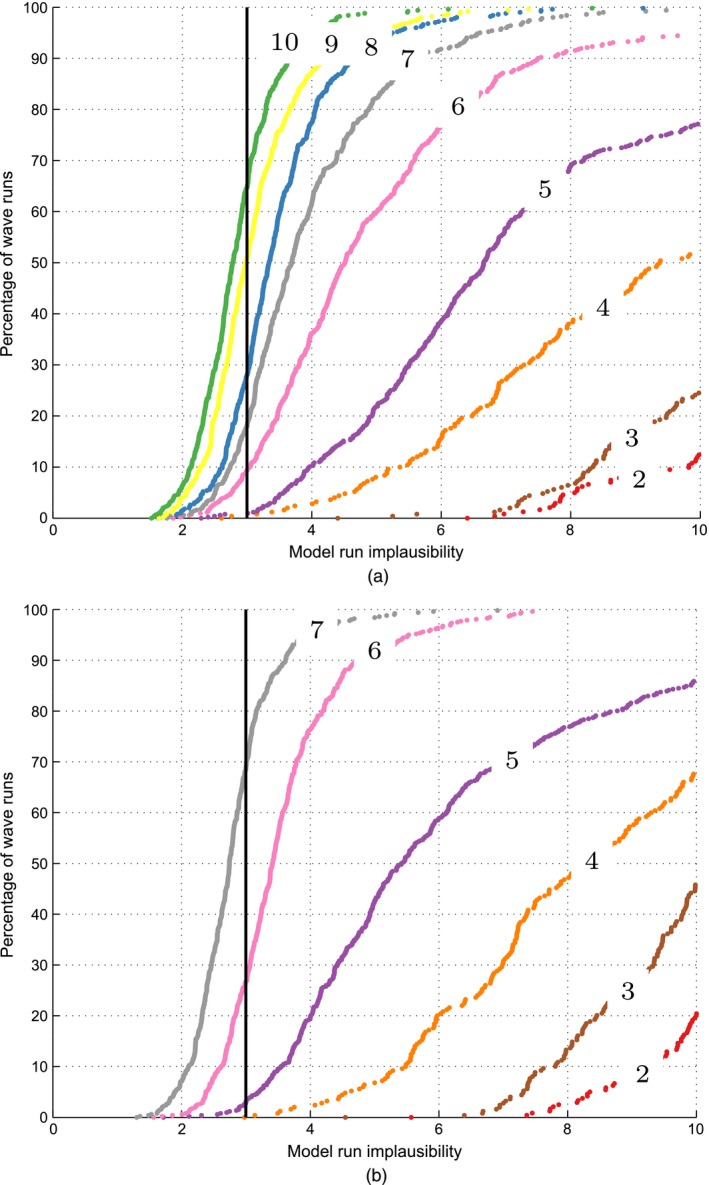
Cumulative distribution function of simulator run implausibility $I_{R}\left(x\right)$, by wave (each curve represents the percentage of each wave's simulator runs with an $I_{R}\left(x\right)$ less than the value indicated by the horizontal axis; the numbers on the curves indicate the wave number): (a) fixed variance; (b) emulated variance

Fig. 3 shows the empirical cumulative distribution of the simulator run implausibility at each wave. Fig. 3 can also be interpreted as the proportion of each wave's simulator runs with an implausibility $I_{R}\left(x\right)$ that is smaller than the value indicated on the horizontal axis. Fig. 3(a) shows the runs from Andrianakis *et al*. (2015) (using the fixed variance approach), and Fig. 3(b) the runs from the methodology proposed. Fig. 3 shows that in Andrianakis *et al*. (2015) the non‐implausible region contained 65% of non‐implausible runs after nine waves. Under the current methodology, the same target was reached after six waves, which is a substantial improvement.

**Figure 4:**
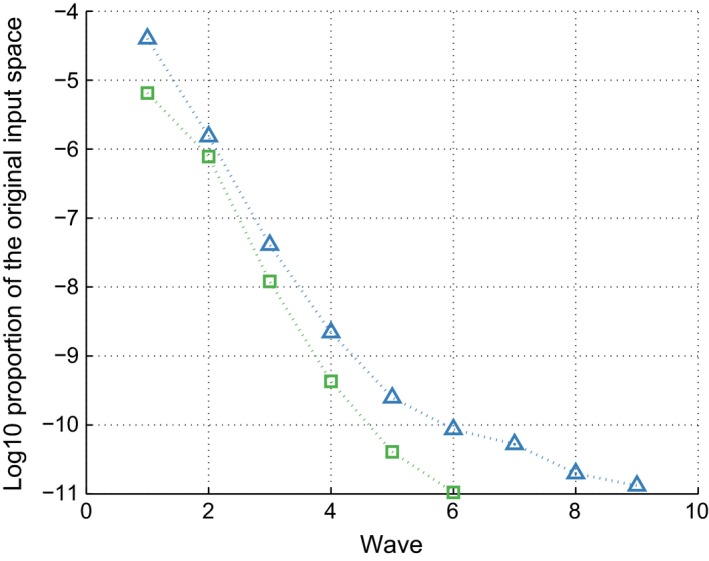
Proportion of the samples drawn at random in the original simulator input space, that are non‐implausible after *k* waves of history matching (the emulated variance methodology proposed achieved the same reduction of non‐implausible space in three fewer waves): 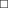, emulated variance; 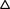, fixed variance

Fig. 4 shows the common logarithmic proportion of the original input space that is calculated as non‐implausible after each wave, using both methodologies. Fig. 4 again shows that the addition of the variance emulators causes the non‐implausible space to shrink by a larger amount at each wave. Especially in later waves, the rate of space reduction is much higher under the approach proposed. The main reason for this is that *s*_*r*_(**x**) dominates the uncertainties in these stages, and having improved estimates from the use of variance emulators allows the space to shrink faster.

### 4.2 Results and insights into the Mukwano simulator

We now discuss the insights that are generated by our analysis of the Mukwano simulator. A practical way of visualizing the reduction of the non‐implausible space over consecutive history matching waves is via the minimum implausibility and optical depth plots. The first type of plot shows, for a grid of values for two selected inputs *x*_1_ and *x*_2_, an estimate of minimum implausibility for an input **x** if we were to fix *x*_1_ and *x*_2_ to a specific value and to vary the remaining components *x*_*i*_, *i*=3,…,*p*. The optical depth plots show an estimate of the probability of obtaining a non‐implausible value if we were to fix *x*_1_ and *x*_2_ to specific values and to sample from the remaining elements of **x** (see Vernon *et al*. (2010a) for details).

**Figure 5:**
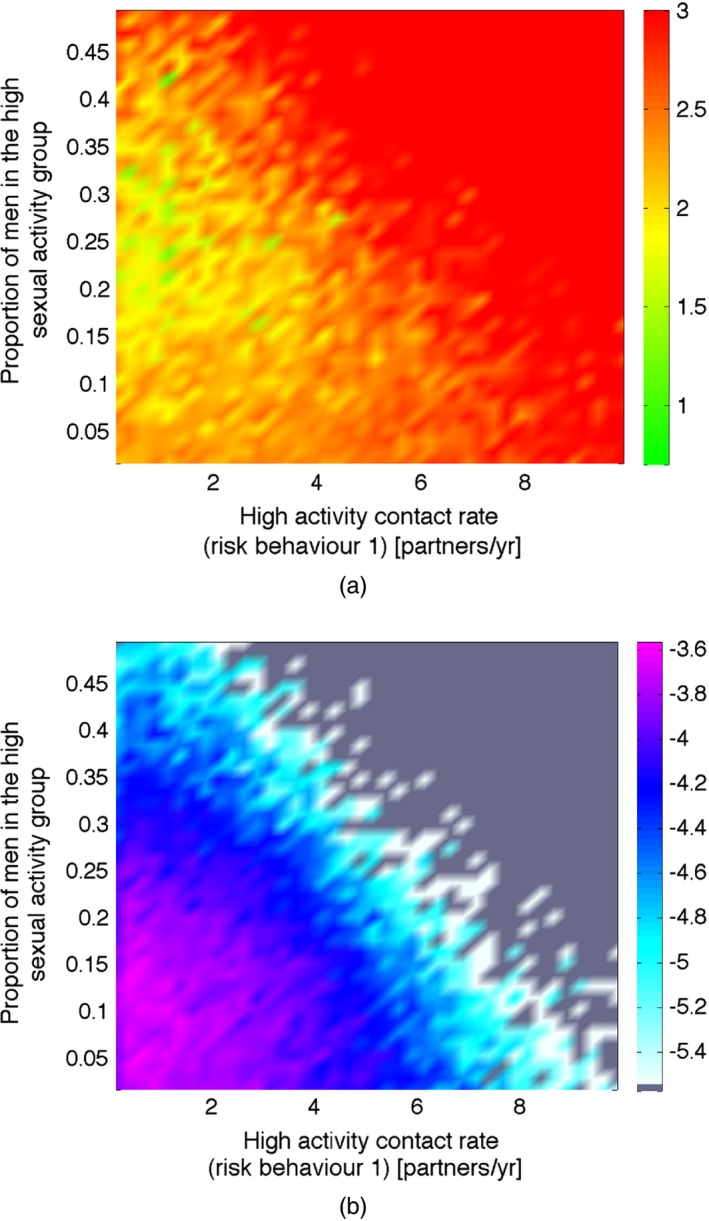
Examples of (a) minimum implausibility and (b) optical depth plots: minimum implausibility plots show an estimate of the minimum implausibility achievable by varying the remaining inputs for different values of the inputs shown along the horizontal and vertical axes; optical depth plots provide an estimate of the common logarithmic probability of finding a non‐implausible point once the two selected inputs have been fixed to a certain value and hence give the depth of the non‐implausible region at each point

Fig. 5 shows an example of these plots for the high activity contact rate (hacr1) and the proportion of men in the high activity group (mhag) inputs. These plots show that, if both inputs take a large value, it is very unlikely that the outputs will match the empirical data, as indicated by the high minimum implausibility value and the low optical depth in the upper right‐hand corners of both figures. This is consistent with behaviour data from the study population in rural Uganda. When both parameters have large values, a high proportion of men will be in the high activity group, and these men will form partnerships at a high rate. This will result in there being too many partnerships in the simulator, and the proportion of men and women with one and/or two or more partnerships will be above the plausible ranges for the associated outputs (outputs 10–18).

The optical depth plots indicate the regions where most of the non‐implausible input space can be found (essentially the depth of the non‐implausible space conditioned on the two inputs that were used for the axes of the plot). In this case, as is shown in Fig. 5(b), this occurs where both hacr1 and mhag are low. This can often be due to a large number of mediocre input points, and therefore a naive search of the input space may be more likely to find solutions within this region. The minimum implausibility plots show the two‐dimensional projection of the regions of input space that can be discarded by different cut‐offs and give an indication, especially in later waves, of where the most promising input points may lie. In this example, this is when hacr1 is low and mhag takes intermediate values, as shown in Fig. 5(a).

**Figure 6:**
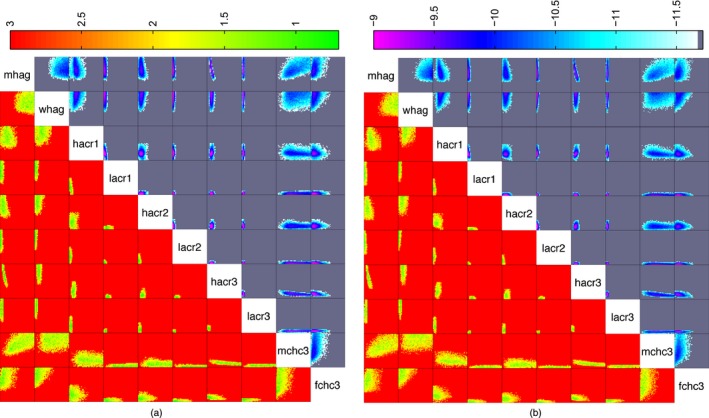
Comparison of the minimum implausibility (below and left of the diagonal) and optical depth plots (above and right of the diagonal) for 10 key inputs (all axes vary between 0 and 1 (normalized); for the minimum implausibility plots, the inputs that appear across the main diagonal vary along the horizontal axis for the plots that appear to the left of the input names, and along the vertical axis for those that appear below; for the optical depth plots, the inputs vary across the horizontal axis for the plots that appear above the input names and across the vertical axis for those that appear to the right): (a) wave 9 (fixed variance); (b) wave 6 (emulated variance)

Fig. 6 shows the combined minimum implausibility and optical depth plots for 10 of the 22 inputs whose range was reduced the most after the history match. Fig. 6(a) shows the rejected space after nine waves by using the fixed variance approach. Fig. 6(b) shows the space reduction after six waves of the methodology proposed. The two‐dimensional projections of the non‐implausible space are very similar, implying that the methodology (emulated variance) achieved a similar input space reduction as the fixed variance approach of Andrianakis *et al*. (2015), in three fewer waves.

Fig. 6 also shows that the male concurrency parameter in the high concurrency group in the third risk period (mchc3) can take higher values when the high activity contact rate in the third risk period (hacr3) is lower. The values of these parameters are constrained by the need to fit the simulator to data on the point prevalence of men with two or more short duration partnerships in 2008 (output 14), which suggest that no more than 2.1% of men have concurrent short duration partnerships at a given point in time. When the high activity contact rate is high, the probability that a man forms a second, concurrent partnership needs to be low, to prevent the point prevalence of concurrent short duration partnerships in the simulator being too high. At lower contact rates, this is relaxed slightly, and the probability that men form additional partnerships can be higher.

**Figure 7:**
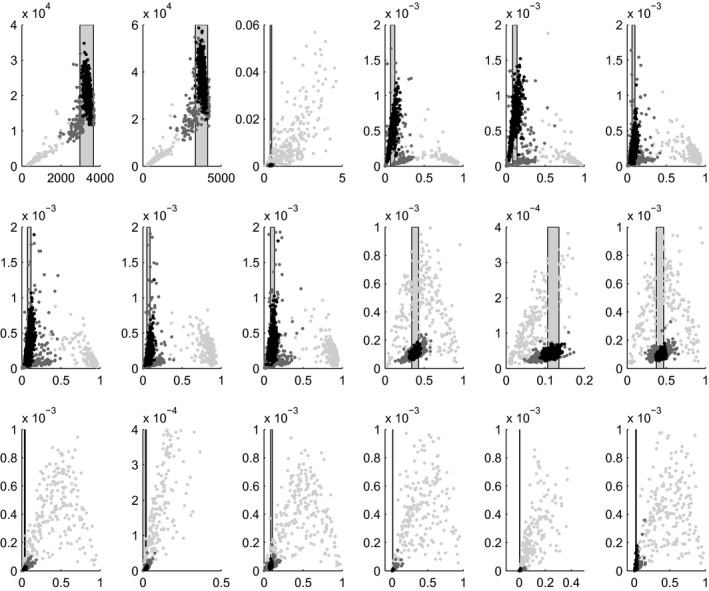
Scatter plots of the simulator's mean output (horizontal axis) against its variance (vertical axis) for waves 1 (), 4 () and 7 (): the 18 outputs are arranged first from left to right and then from top to bottom; the vertical patches in each panel show the empirical data on the mean outputs with ± 2 standard deviations derived from the observation error and model discrepancy at wave 6

Regarding the simulator's outputs Fig. 7 shows 18 panels, one for each output given in Table 2. Each panel shows a scatter plot of the mean against the variance of the simulator runs at each wave. The vertical bands show the empirical data and the associated 2 standard deviations arising from the observation error and model discrepancy at wave 6 and hence represent the target of the history match. Two key conclusions can be extracted from Fig. 7: first, as the history match progresses, the mean output of the simulator converges to the empirical data, as can be seen by the black dots that are centred at the empirical data patches. Second, the variance of the output is far from being constant and varies not only in the first but also in the later waves. This shows that the HIV transmission model has a non‐trivial variance dependence on **x**, even across the tiny region of input space where good matches are to be found. This further justifies the need for estimating the variance, instead of using a fixed and crudely large estimate, as in Andrianakis *et al*. (2015).

**Figure 8:**
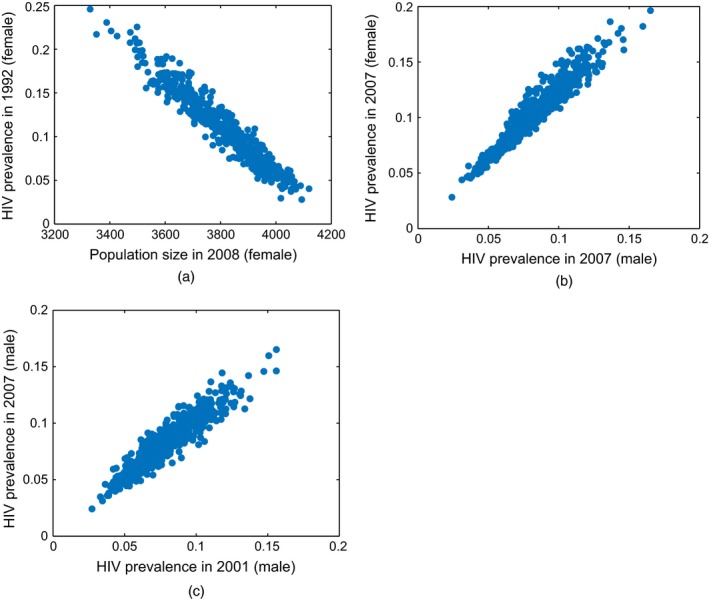
Scatter plots of mean output values from simulator runs at wave 7: the large correlation between outputs is an indication of the way that the simulator models the HIV transmission across sexes and across time (see the text)

Fig. 8 shows scatter plots between simulator outputs at wave 7, which provide insight into how the simulator handles the HIV transmission process. Fig. 8(a) shows a strong negative correlation (*r*=−0.96) between the prevalence of HIV in women in 1992, and the female population size in 2008. This occurs because a high prevalence of HIV in 1992 greatly increases the mortality rate in the simulated population, decreasing population size. As the rate at which new people are born in the simulation is a function of the number of women in the simulated population, this also reduces population growth. Strong positive correlations are present between male and female HIV prevalences in the same year (Fig. 8(b), *r*= 0.96). This occurs because heterosexual sex is the major route of HIV transmission in Uganda and is the only route that is included in the simulator. As simulated men can therefore only be infected by women, and vice versa, male and female HIV prevalences are necessarily highly correlated. There are also strong correlations between HIV prevalences in different years (Fig. 8(c), *r*= 0.94), reflecting the fact that HIV is infectious. This means that the rate of new infections is higher at higher HIV prevalences, and that the prevalence of HIV in any given year is likely to be higher if the prevalence of HIV was also high in earlier years.

### 4.3 Computational cost of the methodology proposed

To allow a comparison of the computational costs between the methodology proposed and the fixed variance approach, the number of simulator runs was kept the same between the two methods at each wave. The numbers of design points per wave are shown in Table 3. The number of design points in the initial waves were approximately 250, following the recommendation of 10 design points per input (Andrianakis *et al*., 2015); this number was doubled from wave 5 onwards to improve the emulators and to increase the space rejection. The simulator was run *K*=100 times at each design point for estimating its mean and variance, which was the same number as in Andrianakis *et al*. (2015) to ensure consistency. The number of emulator evaluations per wave and in total can therefore be extracted by Table 3 by multiplying the quantities by *K*=100.

**Table 3:** Number of design points for which the simulator was run at each wave†

*Wave*	*Fixed*	*Emulated*
	*variance*	*variance*
1	240	240
2	242	242
3	249	249
4	250	250
5	516	516
6	520	520
7	500	—
8	500	—
9	500	—
Total	3517	2017

†The number of simulator evaluations is given by the numbers shown in the table multiplied by *K*=100.

We recorded the times that it took to complete the simulator runs, which are shown in Table 4 in the row ‘Simulator running time’. Note that these calculations assume a 100‐core cluster (which is the average number of cores that we had at our disposal)—running the simulator on a single core, but otherwise identical machine, would have taken 100 times as long. Runs in wave 1 took up to four times longer to complete than the runs in subsequent waves. This was because the simulator run times were longer in some very implausible areas of the input space. This is a common feature of computer models but is more prominent in the stochastic case: we may see vastly different run times in different parts of the input space. Once we were aware of this, say after analysing the wave 1 runs, we could create designs that exploit this feature, but we leave this for future work.

**Table 4:** Total time and breakdown of the tasks involved in history matching Mukwano by using the two approaches†

	*Fixed*	*Emulated*	*% reduction*
	*variance*	*variance*	
Simulator running time (days)	10.5	7.1	32
Emulator training time (days)	1.19	0.41	—
Variance emulator training (days)	—	0.38	—
Total emulator training (days)	1.19	0.79	34
Staff time per wave (h)	4	6	—
Total staff time (days)	1.5	1.5	0
Total time (days)	13.2	9.4	29

†The tasks in the first four rows are parallelizable, and figures assume the usage of a 100‐core cluster (2.5 GHz, 8 Gbytes random‐access memory).

We also recorded the time that was required for training the emulators, noting that, to avoid local minima in the estimation of the emulator's hyperparameters, the optimization routine was initialized from 20 different starting points (see Andrianakis *et al*. (2015) for more details). At each wave, the optimization scheme was run 20*R* times where *R*=18 is the number of outputs, and therefore the number of emulators that we built. Table 4 also shows the time that it took the cluster to run the optimization routines, again assuming the existence of 100 cores. The second row of Table 4 refers to the training of the emulators of the mean, and the third row to the emulators of the variance.

Currently, history matching is not a fully automated procedure, and manual intervention is required from the user at two stages of the process at each wave. The first is to collect the data from the cluster and to set up the emulators to be trained. The second involves using the ‘newly built’ emulators to identify the non‐implausible space, and to select the design points where the simulator is next to be run. Although large parts of this process could be further automated, we believe that manual checks can ensure that the history match is converging to the right values and can save time in the long run. We estimated that approximately 3 h of staff (user) time were required at each of the two stages that were mentioned earlier. For the fixed variance approach, this time was approximately 2 h, as variance emulators did not need to be built.

In summary, and as shown in the last row of Table 4, the use of variance emulators allowed completing the history match around 70% of the time, taking approximately 9.4 days instead of 13.2. The total number of simulator evaluations was also brought down to 201700 from 351700: a 43% reduction.

## 5 Conclusion

In this work, we history matched Mukwano, which is an individual‐based stochastic simulator that models the transmission of HIV in the presence of concurrent relationships. The simulator is used for assessing the effect of concurrent relationships on the incidence and prevalence of the disease and to evaluate this effect relatively to other changes in sexual behaviour. History matching allows calibrating Mukwano to empirical data, which is a step that is necessary before using the simulator to infer any epidemiological parameters or to make predictions about the evolution of the disease.

The present paper addressed a shortcoming of history matching when applied to stochastic models, by explicitly emulating the variance of the outputs, which is an approach that increased the overall efficiency of the method. After six waves, history matching produced samples that matched all Mukwano's 18 outputs 70% of the time, and the parameter space was reduced by a factor of 10^11^. Furthermore, the variance emulation that is proposed here reduced the time required by the method to 70% compared with previous work and also reduced the number of simulator evaluations by 43%.

A study of the non‐implausible space and the calibrated outputs provided useful insights into the simulator's structure. The constraints, imposed by the empirical data, were traced back to the inputs, finding that they created distinctive correlation patterns in the non‐implausible space. For example, constraints in the number of concurrent partnerships meant that contact rates and concurrency parameters could not be simultaneously large or small. Additionally, strong correlations between the simulator's outputs at the last wave illuminated the way that the simulator handles specific aspects of the HIV transmission process. In particular, correlations between HIV prevalence and population size across time and genders revealed the major route of HIV transmission in the simulator and links between HIV prevalence and mortality. This type of analysis enhanced our understanding of Mukwano and can be very helpful in its further development.

Although history matching is a methodology for calibrating slow and high dimensional simulators, such as Mukwano, it is not geared towards making probabilistic statements about the posterior of the simulator's parameters, and instead it should be viewed as

- a useful precalibration step to identify a small region of input space where the posterior will reside (while simultaneously checking that the simulator is fit for purpose, and hence that such a calibration is meaningful) or
- the appropriate analysis for model development and checking of a model that is not thought to be sufficiently accurate to warrant a full Bayesian analysis (see Vernon *et al*. (2010a) and the associated discussions for more details).

In this way, history matching should not be thought of as a direct competitor to other calibration methods, but rather as a procedure that will help to improve the efficiency of whatever subsequent technique we wish to employ. An extension of this method would be to combine it with probabilistic calibration methods, which would typically be computationally infeasible if applied to the original input space of a simulator of Mukwano's complexity but may be successful if they are applied to the greatly reduced non‐implausible space that results from history matching.
